# Personalized Immunotherapy in Osteoarthritis: A Clinical Trial of Autologous Dendritic Cell Immunotherapy in Knee Osteo-Arthritis

**DOI:** 10.3390/cimb48030330

**Published:** 2026-03-20

**Authors:** Kurniawan Silalahi, Bhimo Aji Hernowo, Jonny Jonny, Lintang Sagoro, Chrismis Novalinda Ginting, Terawan Agus Putranto

**Affiliations:** 1 Faculty of Medicine, Dentistry, and Health Sciences, Universitas Prima Indonesia, Medan 20118, Indonesia; karuniaspot@gmail.com (K.S.); jonnyunhan@idu.ac.id (J.J.); chrismis@unprimdn.ac.id (C.N.G.); 2 Department of Orthopaedic and Traumatology, Slamet Riyadi Army Hospital, Surakarta 57148, Indonesia; 3 Faculty of Medicine, Universitas Pembangunan Nasional “Veteran” Jakarta, Jakarta 12450, Indonesia; 4 Indonesia Army Cellcure Center, Gatot Soebroto Central Army Hospital, Jakarta 10410, Indonesia; 5 Faculty of Military Medicine, Indonesia Defense University, Bogor 16810, Indonesia; 6 Nephrology Division, Department of Internal Medicine, Gatot Soebroto Central Army Hospital, Jakarta 10410, Indonesia; 7 Department of Radiology, Gatot Soebroto Army Central Hospital, Jakarta 10410, Indonesia

**Keywords:** dendritic cell, osteoarthritis, knee, immunomodulation

## Abstract

**Background/Objectives**: Osteoarthritis (OA) is a chronic inflammatory disease with limited disease-modifying therapies. This study explored a novel immunomodulatory approach using autologous, antigen-pulsed semi-mature dendritic cells (DCs) to modulate the inflammatory milieu in knee OA patients. **Methods**: In this open-label, quasi-experimental study, 29 subjects received a single subcutaneous injection of autologous DCs. Outcomes assessed at baseline and 4 weeks included the WOMAC index for symptoms and serum levels of IL-6 and TNF-α. Responses were analyzed in the overall cohort and by BMI subgroups. **Results**: The overall cohort showed a non-significant trend in WOMAC improvement (*p* = 0.080) and no change in IL-6 (*p* = 0.785) or TNF-α (*p* = 0.330). Subgroup analysis revealed differential patterns of response: WOMAC scores improved significantly only in normal-weight patients (*p* = 0.030), while serum TNF-α decreased significantly only in overweight patients (*p* = 0.025). IL-6 levels were unchanged across all groups. **Conclusions**: Autologous antigen-pulsed DC administration was associated with differential responses across BMI subgroups. Symptomatic benefit was observed in normal-weight individuals, while a reduction in systemic TNF-α occurred in overweight patients. These findings suggest that the host metabolic state may modulate the response to DC-based immunotherapy, and therefore warrant validation in a randomized, placebo-controlled trial.

## 1. Introduction

Osteoarthritis (OA) of the knee remains a pervasive and debilitating condition, characterized by the progressive degradation of articular cartilage, synovial inflammation, and subchondral bone remodeling. Current mainstream therapeutic strategies, including pharmacologic management with NSAIDs and corticosteroids, physical therapy, and ultimately joint replacement surgery, are largely palliative or invasive, failing to address the underlying pathophysiological drivers of disease progression [1]. This significant unmet clinical need has catalyzed the exploration of disease-modifying interventions, with recent attention turning toward the pivotal role of the immune system in OA pathogenesis.

Within the osteoarthritic joint, the delicate balance between pro-inflammatory and anti-inflammatory signals is profoundly disrupted, fostering a state of chronic, low-grade inflammation that perpetuates tissue damage [2]. In this immunologic context, dendritic cells (DCs) emerge as critical orchestrators. As master antigen-presenting cells, DCs are essential not only for initiating immune responses but also for regulating them, including the induction and maintenance of immunological tolerance [3]. Their strategic position makes them attractive therapeutic vectors; by modulating DC function, it may be possible to skew the local and systemic immune milieu from a destructive, inflammatory state toward a protective, anti-inflammatory one. Preclinical evidence supports this concept, demonstrating that DCs engineered to promote regulatory T cell (Treg) responses can mitigate inflammation and improve joint conditions in models of OA [4,5]. Early clinical reports further suggest that autologous DC transfer is feasible and may offer a favorable safety profile with indications of symptomatic relief [6,7].

However, the clinical translation of DC therapy for OA is in its infancy, constrained by several challenges. Existing studies are predominantly preclinical or involve small, heterogeneous patient cohorts, limiting definitive conclusions about efficacy [8,9]. The technical complexity of cell manufacturing and a lack of standardized protocols also present significant hurdles [10]. Most critically, the optimal antigenic stimulus to program DCs for a robust and clinically meaningful anti-OA effect remains undefined. The choice of antigen is fundamental, as it determines the specificity and quality of the subsequent immune modulation.

The present study employs an autologous DC preparation platform originally developed for personalized COVID-19 vaccination [11]. In this system, monocyte-derived DCs are pulsed with recombinant SARS-CoV-2 spike protein ex vivo. Importantly, the spike protein in this context serves not as a disease-specific antigen for OA, but rather as a defined maturation stimulus designed to drive DCs toward a semi-mature state. Semi-mature DCs—characterized by intermediate expression of co-stimulatory molecules and a cytokine profile favoring anti-inflammatory mediators such as IL-10 over pro-inflammatory IL-12—have been extensively studied as potential inducers of immune regulation in autoimmune and inflammatory conditions [12]. The generation of semi-mature DCs through controlled antigen exposure has been shown in preclinical models to promote regulatory T cell expansion and suppress effector T cell responses, thereby shifting the immune balance toward tolerance [13]. We hypothesized that this semi-mature DC phenotype, once administered to patients with OA, could exert immunomodulatory effects on the chronic inflammatory milieu of the osteoarthritic joint, independent of the specific identity of the pulsing antigen.

To address this approach, we conducted a clinical observation study administering subcutaneous injections of autologous semi-mature dendritic cells to patients with knee OA across the spectrum of radiographic severity. Outcomes were assessed using the Western Ontario and McMaster Universities Osteoarthritis Index (WOMAC) and by measuring serum levels of IL-6 and TNF-α.

## 2. Materials and Methods

### 2.1. Study Design and Ethical Approval

This was an open-label, quasi-experimental clinical observation study (ClinicalTrials.gov identifier: NCT06866158). The study protocol was reviewed and approved by the Ethics Committee of the Gatot Soebroto Army Hospital (Ethical Clearance No. 126/XI/KEPK/2024, dated 7 November 2024). The study was conducted in accordance with the principles of the Declaration of Helsinki. All participants provided written informed consent prior to enrollment. Patient recruitment and follow-up took place between November 2024 and January 2025.

### 2.2. Patient Recruitment and Eligibility

Participants were recruited from the internal medicine outpatient clinic of Gatot Soebroto Army Hospital. Eligible subjects were adults over 50 years of age with a radiographic diagnosis of knee osteoarthritis, as defined by the Kellgren–Lawrence (KL) grading system on standing anteroposterior knee X-rays, which were assessed at baseline for diagnostic confirmation. General good physical and mental health, including normal to moderate obesity (Body Mass Index, BMI, <25–40 kg/m^2^), was required as per investigator assessment.

Key exclusion criteria included: recent use (within 4 weeks) of immunosuppressive therapy (e.g., corticosteroids, hydroxychloroquine, methotrexate); positive pregnancy test; known immunodeficiency diseases (e.g., HIV, HCV, HBV); a condition requiring supplemental oxygen; diagnosis of invasive cancer with ongoing anticancer therapy (except hormonal therapy for breast or prostate cancer); a history or genetic predisposition to thromboembolism with concomitant anticoagulant therapy (excluding low-dose aspirin); or any physical or mental disability hindering normal daily activities. Subjects were also excluded for uncontrolled hypertension (systolic > 180 mmHg, diastolic > 100 mmHg), severe obesity (BMI > 40 kg/m^2^), or any other acute or chronic medical condition deemed by the investigator to pose a risk, interfere with protocol adherence, or complicate evaluation.

### 2.3. Dendritic Cell Preparation and Administration

For each participant, 40 mL of peripheral blood was collected via venipuncture. Peripheral blood mononuclear cells (PBMCs) were isolated by density-gradient centrifugation, and monocytes were differentiated into immature dendritic cells (DCs) during a 5-day culture in medium supplemented with recombinant human granulocyte-macrophage colony-stimulating factor (GM-CSF) and interleukin-4 (IL-4). To generate a semi-mature, immunomodulatory phenotype, the immature DCs were pulsed ex vivo with recombinant pre-fusion stabilized SARS-CoV-2 spike (S) protein ectodomain with a C-terminal His tag, furin site removed, and stabilized trimer. All cell processing reagents, culture media, and the recombinant spike protein antigen were supplied by AIVITA Biomedical (Irvine, CA, USA) as a proprietary, custom-manufactured dendritic cell preparation kit. Because the kit is manufactured as a bespoke research-grade product tailored to this specific DC preparation protocol, conventional commercial catalog numbers are not assigned. After a 2-day incubation with the antigen, the DCs were harvested, washed, and resuspended in sterile saline. The entire yield of antigen-pulsed, autologous DCs derived from the initial 40 mL blood volume was administered as a single subcutaneous injection in the left upper arm and monitored for any immediate adverse event within 60 min after injection. The administered cell dose was therefore dependent on individual yield, typically ranging between 0.5 × 10^6^ and 8 × 10^6^ cells. This DC preparation protocol follows a patient-specific, yield-dependent dosing approach rather than a fixed cell-number paradigm. The antigen pulsing concentration (0.1 µg recombinant spike protein) was standardized across all patients. This approach was adopted from the parent vaccine platform [11], in which dose–response characterization was performed during Phase I/II evaluation. As the present study utilized the same manufacturing process without modification, individual dose-outcome correlation analysis was not performed.

### 2.4. Quality Control and Release Criteria

Cell viability and concentration of the final DC product were assessed prior to administration using the CASY Cell Counter and Analyzer System (OmniLife, Bremen, Germany). A 50 µL aliquot of the final cell suspension was diluted in 10 mL of CASYton solution and analyzed per the manufacturer’s protocol. A minimum viability threshold of ≥90% was required for product release; batches failing this criterion were discarded and the participant was rescheduled. Endotoxin testing was not performed on each individual final cell product because cell processing was conducted in a certified ISO Class 7 [14] cleanroom facility at the Indonesia Army Cellcure Center under current Good Manufacturing Practice (cGMP)-compliant conditions following applicable guidelines and regulations.

### 2.5. Clinical and Biochemical Assessments

Clinical and biochemical evaluations were performed at baseline and four weeks post-intervention. Disease-specific pain and function were evaluated using the validated Indonesian version of the Western Ontario and McMaster Universities Osteoarthritis Index (WOMAC) Likert 3.1. Only the total WOMAC score was analyzed in this study; individual subscale scores (pain, stiffness, physical function) were not evaluated separately, as the study was designed to assess overall symptomatic change in this exploratory cohort. Venous blood samples were collected at both time points. Serum was separated by centrifugation and stored at −80 °C until batch analysis. Serum concentrations of interleukin-6 (IL-6) and tumor necrosis factor-alpha (TNF-α) were quantified in duplicate using commercially available enzyme-linked immunosorbent assay (ELISA) kits (Reed Biotech Ltd., Wuhan, China), according to the manufacturer’s instructions. All samples from the 29 enrolled subjects were processed in a single assay run to minimize inter-assay variability.

### 2.6. Safety Assessment

Safety was monitored through structured clinical assessments at each study visit. Adverse events (AEs) were recorded and graded according to the Common Terminology Criteria for Adverse Events (CTCAE) version 5.0. Local injection-site reactions (erythema, swelling, pain, induration) and systemic reactions (fever, fatigue, myalgia) were specifically solicited within 7 days post-injection. Routine laboratory safety parameters, including complete blood count, renal function (serum creatinine, blood urea nitrogen), and liver function (ALT, AST), were assessed at baseline and at the 4-week follow-up visit. Any serious adverse event (SAE), defined as any event resulting in death, hospitalization, disability, or requiring medical intervention to prevent a serious outcome, was to be reported to the ethics committee within 24 h.

### 2.7. Statistical Analysis

A priori sample size calculation, based on an expected change in WOMAC score with an alpha of 0.05 and power of 0.8, indicated a minimum requirement of 27 participants. Data normality was assessed using the Shapiro–Wilk test. For comparisons of continuous demographic variables between groups, paired *t*-tests or Wilcoxon signed-rank tests were applied as appropriate. All analyses were performed using IBM SPSS Statistics software, version 25.0. Given the exploratory nature of this study and the small sample size, no correction for multiple comparisons was applied. Accordingly, all subgroup results should be interpreted to answer hypotheses rather than being confirmatory.

### 2.8. Outcome Measures Reported in This Study

This trial was registered at ClinicalTrials.gov (NCT06866158). The primary outcome measures specified in the registration have been reported in a separate publication. The DC preparation protocol was adopted without modification from our previously published work [15]. What was specifically adapted for the current study were the clinical outcome measures: change in total WOMAC score from baseline to 4 weeks post-intervention, and changes in serum IL-6 and TNF-α concentrations over the same period. Immunogenicity endpoints (e.g., antigen-specific T-cell or antibody responses) were not evaluated in this study, as the objective of this report was to assess clinical and anti-inflammatory outcomes rather than adaptive immune priming. The ELISPOT and anti-RBD ELISA methods described in the original DC vaccine platform protocol [11] were not performed in this OA trial.

### 2.9. Generative AI Disclosure

During the preparation of this work, the authors used DeepSeek AI version 3.2 to assist with language editing and refinement for readability. After using this tool, the authors reviewed and edited the content as needed and take full responsibility for the content of the published work.

## 3. Results

### 3.1. Patient Demographics and Baseline Characteristics

A total of 29 patients with knee osteoarthritis were enrolled and completed the study. The cohort comprised 8 men (27.6%) and 21 women (72.4%), with a mean age of 63 years (range 40–76). The vast majority (96.6%) of enrolled subjects had concomitant hypertension. Other common comorbidities included neuropathy (65.5%) and cardiac disease (51.7%). According to body mass index (BMI) categories, 10 patients (34.5%) were of normal weight (BMI < 25 kg/m^2^), 9 patients (31.0%) were overweight (BMI 25–30 kg/m^2^), and 10 patients (34.5%) were obese (BMI ≥ 30 kg/m^2^). Baseline characteristics of the study cohort are summarized in Table 1.

### 3.2. Safety

The autologous DC injection was well tolerated in all 29 participants. No serious adverse events (SAEs) were reported during the study period. The most common adverse events were mild, self-limiting injection-site reactions (grade 1 per CTCAE v5.0), including transient erythema (*n* = 3, 10.3%) and mild pain at the injection site (*n* = 5, 17.2%), all resolving within 48 h without intervention. No systemic reactions (fever, fatigue, or allergic reactions) were observed. Routine laboratory safety parameters (complete blood count, renal function, and liver function) showed no clinically significant changes from baseline to 4-week follow-up in any participant.

### 3.3. Clinical Outcomes: WOMAC Score

The pre-specified clinical outcome, total WOMAC score, showed a non-significant trend towards improvement in the overall cohort four weeks post-intervention (median: 22.9 pre-treatment vs. 19.7 post-treatment, *p* = 0.080). Subgroup analysis based on baseline BMI revealed a statistically significant improvement exclusively in the normal-weight group (BMI < 25 kg/m^2^), where the median WOMAC score decreased from 22.9 to 9.3 (*p* = 0.030). No significant within-group changes were observed in the overweight (BMI 25–30 kg/m^2^) or obese (BMI ≥ 30 kg/m^2^) subgroups (*p* = 0.188 and *p* = 0.249, respectively). The results are detailed in Table 2.

### 3.4. Interleukin-6

No significant changes in serum IL-6 levels were observed following dendritic cell administration in the overall cohort (median: 3.75 pg/mL pre vs. 4.72 pg/mL post, *p* = 0.785) or in any BMI-defined subgroup (all *p* > 0.05). A near-significant decrease was noted in the overweight subgroup (*p* = 0.051). Results are shown in Table 3.

### 3.5. Tumor Necrosis Factor-Alpha

Analysis of the entire cohort showed no significant change in TNF-α levels: median 2.16 pg/mL pre vs. 2.01 pg/mL post, *p* = 0.330. However, a distinct response pattern emerged upon BMI stratification. A significant reduction in TNF-α was observed in the overweight subgroup (BMI 25–30 kg/m^2^), with mean levels decreasing from 2.21 ± 0.61 pg/mL to 1.93 ± 0.47 pg/mL (*p* = 0.025). In contrast, no significant changes were found in the normal-weight or obese subgroups (*p* = 0.721 and *p* = 0.540, respectively). The biomarker results are consolidated in Table 4.

## 4. Discussion

This clinical observation investigated the effects of autologous, antigen-pulsed dendritic cell administration on clinical symptoms and systemic inflammation in patients with knee osteoarthritis. The present analysis focuses on WOMAC, IL-6, and TNF-α. The principal finding is a distinct dissociation in observed response based on patient BMI. Symptomatic improvement, measured by the WOMAC score, was significant only in normal-weight individuals, while a reduction in the systemic pro-inflammatory cytokine TNF-α was observed exclusively in the overweight subgroup. This pattern suggests that the immunomodulatory action of this therapy may engage different biological pathways depending on the patient’s metabolic state.

The significant improvement in WOMAC score within the normal-weight subgroup aligns with established literature linking lower BMI to better functional outcomes in OA management. Studies have consistently shown that individuals with a normal BMI demonstrate greater improvements in pain and physical function scores following various interventions [16]. Furthermore, targeted dietary interventions leading to BMI reduction are associated with significant decreases in WOMAC scores, underscoring the role of metabolic health in functional improvement [17]. Our finding suggests that in the absence of significant obesity-related meta-inflammation, the immunomodulatory signal from dendritic cells may more effectively translate into perceptible clinical benefit, possibly by modulating local joint inflammation without the confounding burden of systemic adipokine-driven pathology.

Phenotypic characterization of the DC product was not performed in this study, and the immunomodulatory mechanism underlying the observed biomarker changes undetermined. The DC preparation used here was monocyte-derived DCs differentiated with GM-CSF and IL-4, then pulsed with recombinant spike protein which was originally developed as an immunogenic vaccine platform for COVID-19 prevention [11]. The observation that this DC product appears to exert anti-inflammatory effects when administered to patients with a chronic inflammatory condition is unexpected and currently unexplained. However, preclinical evidence suggests that GM-CSF/IL-4 differentiation alone, without pharmacological tolerizing agents (e.g., dexamethasone, vitamin D3), can confer immunomodulatory capacity to monocyte-derived DCs, including Treg expansion and disease inhibition in a type 1 diabetes mouse model [18]. Whether the DCs administered in this trial adopted a tolerogenic or alternative immunomodulatory phenotype, and whether this is causally linked to the observed TNF-α reduction and WOMAC improvement, cannot be determined from the present data. Future studies should incorporate flow cytometric characterization of the DC product (CD80, CD86, CD83, HLA-DR, PD-L1), DC supernatant cytokine profiling (IL-10, IL-12, TGF-β), and quantification of circulating regulatory T cells to elucidate the mechanism of action.

Conversely, the significant reduction in serum TNF-α specifically in the overweight subgroup points to a targeted effect on a key mediator of obesity-associated inflammation. Adipose tissue in overweight and obese individuals is a known source of chronic, low-grade systemic inflammation, characterized by elevated levels of pro-inflammatory cytokines such as TNF-α [19,20]. This inflammatory milieu exacerbates joint degeneration and pain in OA. The observed decrease in TNF-α suggests that dendritic cell administration may exert a modulatory effect on this specific adipose-driven inflammatory pathway. This aligns with therapeutic strategies targeting TNF-α, which have been shown to reduce inflammatory markers and symptoms in OA contexts [21,22]. The lack of a corresponding WOMAC improvement in this group raises the possibility that the reduction in systemic TNF-α, while biologically meaningful, may be insufficient to override other drivers of pain and functional limitation in overweight patients over a 4-week period, or that pain perception in this group is mediated by factors beyond this single cytokine.

The dissociation between symptomatic (WOMAC) and inflammatory (TNF-α) responses suggests a pathophysiological divergence based on metabolic status. In normal-weight patients, OA may be driven predominantly by localized joint immune dysregulation, which the dendritic cell therapy may have ameliorated, leading to significant WOMAC improvement as it potentially addressed the primary source of symptoms [23,24]. Conversely, in overweight patients, OA pathology is superimposed on a state of systemic meta-inflammation fueled by adipose tissue [25,26]. Here, the therapy appeared to reduce this systemic inflammatory burden (lowering TNF-α), but this short-term biochemical correction was insufficient to reverse the cumulative structural and sensory sequelae within the joint, explaining the lack of concurrent symptomatic relief [27]. This divergence highlights the potential for personalized OA management tailored to the dominant pathophysiology—localized immune dysregulation versus systemic metabolic inflammation.

The absence of a significant change in IL-6 levels across all groups is notable. While IL-6 is a well-established player in OA pathogenesis, implicated in synovitis, cartilage degradation, and pain [28,29], our intervention did not alter its systemic concentration. This may indicate that the immunomodulatory mechanism of action of these particular dendritic cells is more selectively tuned to pathways regulating TNF-α, or that effects on IL-6 are more localized to the joint environment and not reflected in peripheral blood over this short timeframe.

This study has several limitations. First and foremost, its open-label, single-arm design and the absence of a control group preclude definitive conclusions about efficacy and make it impossible to rule out placebo effects or natural history, particularly for the WOMAC score. The follow-up period was short (4 weeks), limiting assessment of durability.

Furthermore, the study cohort had significant metabolic comorbidities, most notably widespread hypertension, which may influence both baseline inflammation and treatment response, potentially limiting the generalizability of findings to a healthier OA population. Despite these limitations, the identified biomarker response (TNF-α reduction in overweight) provides a compelling biological signal that warrants validation in a randomized, placebo-controlled trial with longer follow-up and integrated local joint imaging.

## Figures and Tables

**Table 1:** Baseline Characteristics of the Study Cohort.

Characteristics		*n*
Sex	Men	8
	Women	21
Age, range (mean)	40–76 (63)	
	<60	5
	>60	24
Body Mass Index	Underweight	0
	Normal weight	10
	Overweight	9
	Obese	10
Comorbidity	Hypertension	28
	Neuropathy	19
	Cardiac Disease	15
	Dyslipidemia	13
	Hyperuricemia	8

**Table 2:** WOMAC Outcomes Before and After Dendritic Cell Immunotherapy.

Group	*n*	Pre-Treatment Median (IQR)	Post-Treatment Median (IQR)	*p*-Value
Overall	29	22.9 (12.3–32.7)	19.7 (4.2–24.5)	0.080
Men	8	19.2 (2.1–30.9)	10.4 (2.1–19.8)	0.310
Women	21	22.9 (15.6–38.5)	23.9 (6.8–28.6)	0.500
Normal weight	10	22.9 (15.8–34.4)	9.3 (2.9–27.9)	0.030 *
Overweight	9	14.5 (1.6–32.3)	19.7 (14.1–27.1)	0.188
Obese	10	24.9 (11.2–43.5)	20.8 (10.9–24.2)	0.249

* *p* < 0.05. Statistical test: Wilcoxon signed-rank test or paired *t*-test as appropriate based on Shapiro–Wilk normality assessment for each subgroup (see Section 2.6). IQR = interquartile range.

**Table 3:** Interleukin-6 Outcomes Before and After Dendritic Cell Immunotherapy.

Group	*n*	Pre-Treatment Median (IQR)	Post-Treatment Median (IQR)	*p*-Value
Overall	29	3.75 (2.6–4.3)	4.72 (2.4–6.8)	0.785
Men	8	3.17 (2.1–3.8)	3.40 (1.6–4.8)	0.575
Women	21	3.78 (3.1–5.2)	5.08 (2.5–6.9)	0.852
Normal weight	10	3.17 (2.4–4)	5.24 (3.1–8.6)	0.173
Overweight	9	2.82 (2.24–4.74)	2.16 (2.1–3.9)	0.051
Obese	10	4.06 (3.6–5.9)	5.13 (2.4–6.5)	0.799

Statistical test: Wilcoxon signed-rank test (IL-6 data were non-normally distributed per Shapiro–Wilk test). IQR = interquartile range.

**Table 4:** Tumor Necrosis Factor-alpha Outcomes Before and After Dendritic Cell Immunotherapy.

Group	*n*	Pre-Treatment Median (IQR)	Post-Treatment Median (IQR)	*p*-Value
Overall	29	2.16 (1.8–2.7)	2.01 (1.8–2.6)	0.330
Men	8	2.44 (1.9–2.7)	2.39 (1.8–2.6)	0.575
Women	21	2.07 (1.7–2.6)	1.89 (1.8–2.7)	0.520
Normal weight	10	2.14 (2–2.5)	1.93 (1.8–2.8)	0.721
Overweight *	9	2.21 ± 0.61	1.93 ± 0.47	0.025
Obese	10	2.61 (1.5–2.8)	2.57 (1.8–3)	0.540

Statistical test: Wilcoxon signed-rank test (TNF-α data were non-normally distributed per Shapiro–Wilk test). IQR = interquartile range. * overweight group data were normally distributed, data shown as mean ± SD, hypothesis test using paired *t*-test.

## Data Availability

The data presented in this study are available on request from the corresponding author due to privacy and ethical restrictions to ensure subject’s personal information.

## Abbreviations

The following abbreviations are used in this manuscript:

BMI	Body Mass Index
DCs	Dendritic Cells
ELISA	Enzyme-Linked Immunosorbent Assay
GM-CSF	Granulocyte-Macrophage Colony-Stimulating Factor
IL-4	Interleukin-4
IL-6	Interleukin-6
KL	Kellgren–Lawrence (grading system)
OA	Osteoarthritis
PBMCs	Peripheral Blood Mononuclear Cells
TNF-α	Tumor Necrosis Factor-alpha
Treg	Regulatory T Cell
WOMAC	Western Ontario and McMaster Universities Osteoarthritis Index
